# Maternal versus artificial rearing shapes the rumen microbiome having minor long‐term physiological implications

**DOI:** 10.1111/1462-2920.14801

**Published:** 2019-10-08

**Authors:** Alejandro Belanche, David R. Yáñez‐Ruiz, Andrew P. Detheridge, Gareth W. Griffith, Alison H. Kingston‐Smith, Charles J. Newbold

**Affiliations:** ^1^ Estacion Experimental del Zaidín (CSIC) Profesor Albareda, 1, 18008 Granada Spain; ^2^ IBERS Aberystwyth University SY23 3DA Aberystwyth UK; ^3^ SRUC, Peter Wilson Building, King's Buildings EH9 3JG Edinburgh UK

## Abstract

Increasing productivity is a key target in ruminant science which requires better understanding of the rumen microbiota. This study investigated how maternal versus artificial rearing shapes the rumen microbiota using 24 sets of triplet lambs. Lambs within each sibling set were randomly assigned to natural rearing on the ewe (NN); ewe colostrum for 24 h followed by artificial milk feeding (NA); and colostrum alternative and artificial milk feeding (AA). Maternal colostrum feeding enhanced VFA production at weaning but not thereafter. At weaning, lambs reared on milk replacer had no rumen protozoa and lower microbial diversity, whereas natural rearing accelerated the rumen microbial development and facilitated the transition to solid diet. Differences in the rumen prokaryotic communities disappear later in life when all lambs were grouped on the same pasture up to 23 weeks of age. However, NN animals retained higher fungal diversity and abundances of *Piromyces*, *Feramyces* and Diplodiniinae protozoa as well as higher feed digestibility (+4%) and animal growth (+6.5%) during the grazing period. Nevertheless, no correlations were found between rumen microbiota and productive outcomes. These findings suggest that the early life nutritional intervention determine the initial rumen microbial community, but the persistence of these effects later in life is weak.

## Introduction

Ruminants are unique among livestock species in that they convert non‐human edible forages to human‐edible protein. This is due to the rumen, a foregut fermentation chamber housing a great diversity of bacteria, methanogens, protozoa and fungi which interact, both symbiotically and competitively, to ferment dietary fibre to meet the energy and protein requirements of the host. Consequently, these multi‐kingdom interactions within the rumen have a large effect on ruminant productivity (Dehority, 2003). Due to the restrictions on the use of antibiotics as growth promoters, there have been significant efforts during the last decades to develop novel nutritional strategies and feed additives (i.e. saponins, tannins, essential oils and methane analogues), aiming to shift the rumen fermentation towards more efficient metabolic pathways (Patra, 2010). However, the effectiveness of such strategies appears limited and the results of these interventions are often inconsistent or short‐lived due to high microbial redundancy (overlap of function among multiple species) and resilience (resistance to, and capacity to recover from, perturbation) in the rumen (Weimer, 2015). These factors make it difficult to modify a well established and fully matured microbial ecosystem in the rumen of adult animals (Zhou *et al*., 2018).

The developing rumen in the newborn animal may provide a unique opportunity for the manipulation of such complex microbial ecosystem (Yáñez‐Ruiz *et al*., 2015). Several studies have suggested that it is possible to modify the pattern of rumen microbial colonization in young animals through different early life nutritional interventions (Yañez‐Ruiz *et al*., 2010, Abecia *et al*., 2017), but there is still a general lack of understanding of the mechanism governing these processes such as host–genetics or microbial interactions.

Two main systems exist for rearing offspring in ruminant production: in commercial dairy systems, newborns are generally separated from their dams within the first hours of life and artificially reared with colostrum alternatives and milk replacer; conversely, in meat production systems, newborn animals commonly remain with their dams until weaning (natural rearing). It has been suggested that the nature of the colostrum and milk received by the newborn animals could modulate the rumen microbial colonization (Yeoman *et al*., 2018), but more studies are needed to determine the persistence of such effects later in life (Dill‐McFarland *et al*., 2017; Dill‐McFarland *et al*., 2019).

In a previous paper, we demonstrated that the use of colostrum alternatives and milk replacer can facilitate the artificial rearing of healthy lambs but lead to lower productivity performance in grazing systems (Belanche *et al*., 2019aa). Here, we hypothesize that the type of rearing during early life may shape the rumen microbiota, causing short‐ and long‐lasting effects on the rumen function and animal productivity. The use of a large number of experimental animals allowed us to implement a systems biology approach to describe the whole rumen microbiota in terms of community structure, abundance, diversity and core microbiota of the main microbial groups. Moreover, sibling lambs were used to provide similar genetic background and prenatal environment across treatments.

## Results

### *Animal performance and rumen fermentation*

This study investigated the effects of three different rearing strategies based on (i) natural rearing on the ewe (**NN**), (ii) ewe colostrum for 24 h followed by artificial milk feeding (**NA**) and (iii) the use of colostrum alternative immediately after birth followed by artificial milk feeding (**AA**). A total 24 sets of triplet lambs were used, with one lamb from each sibling set randomly assigned to each rearing system. To elucidate the short‐ and long‐term effects of these rearing strategies on the rumen microbiota, animals were sampled at weaning (6 weeks of age) and after a grazing period in which all lambs and their dams grazed on the same pasture (23 weeks of age).

**Figure 1:**
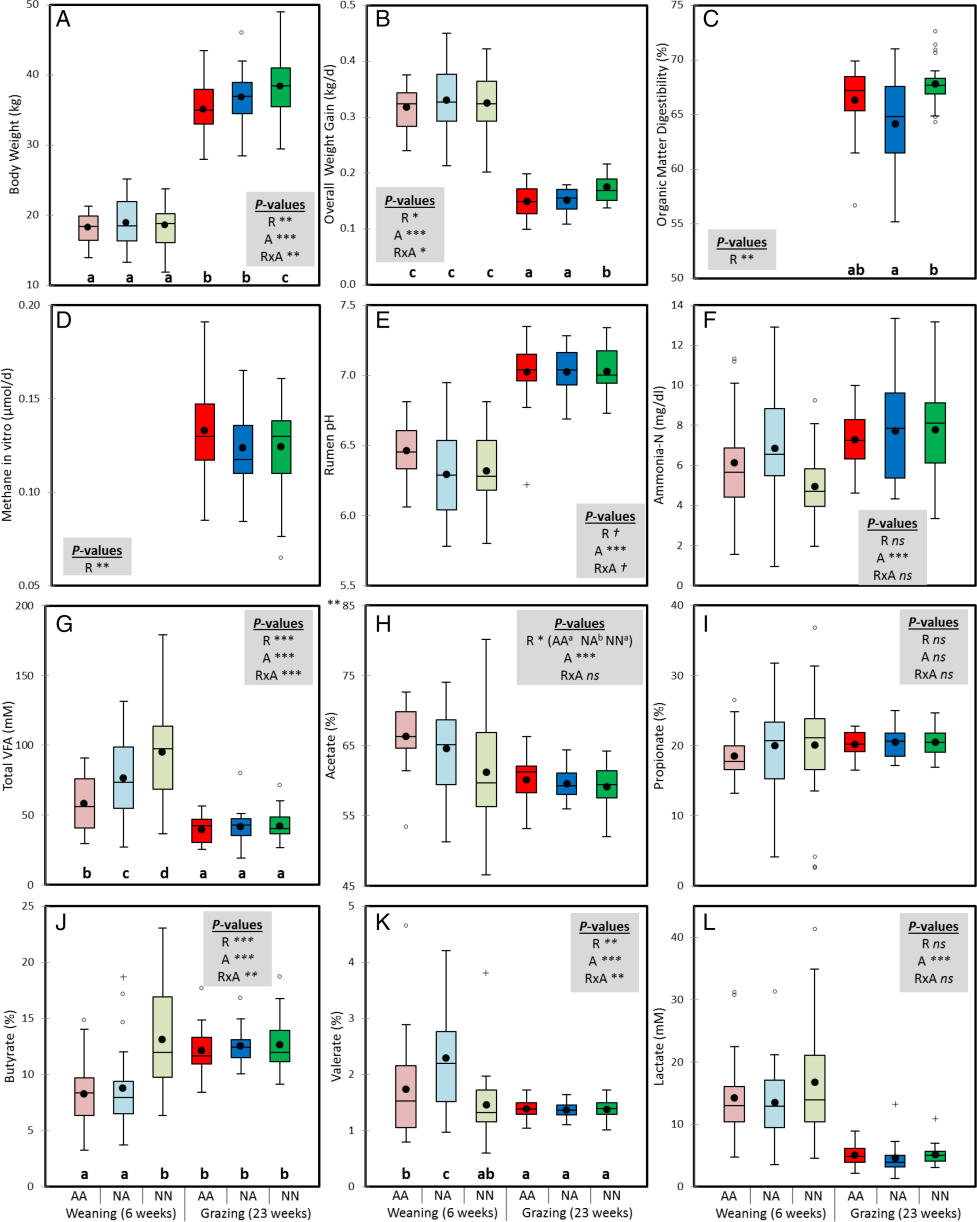
Boxplot indicating the short‐ (6 weeks of age) and long‐term effects (23 weeks) of the rearing system on animal performance and rumen fermentation parameters in lambs. Treatments: AA, colostrum alternative and artificial milk feeding, NA, ewe colostrum and artificial milk feeding; NN, natural rearing. ***p < 0.001; **p < 0.01; *p < 0.05; †*p* < 0.1; ns, not significant for the effects of rearing (R), age (A) and interaction (R × A). Boxes without a common letter differ based on a significant interaction. [Color figure can be viewed at http://wileyonlinelibrary.com]

Under our experimental conditions, the effect of the animal's sex on the rumen fermentation and microbiota was negligible (*p* > 0.1) and thus it is not further discussed. Average creep feed intakes prior to weaning were 96, 137 and 256 g of dry matter (DM) per animal and day for treatments AA, NA and NN respectively. Significant interactions between rearing system and age (R × A) were noted in terms of animal performance (Fig 1A–C): at weaning, all treatments showed similar body weights, but at 23 weeks of age, NN lambs were heavier (mean ± standard error, 38.6 ± 5.2 vs 36.2 ± 4.4 kg, interaction *p* = 0.01) as a result of their higher average daily gain (ADG, 176 ± 26 vs 151 ± 27 g/d, interaction *p =* 0.05) and total tract organic matter (OM) digestibility (67.7 ± 2.1 vs 65.1 ± 3.7%, *p* = 0.001) compared to their artificially reared counterparts (AA and NA). As a result, the rearing system explained only a small proportion of the variance for the ADG before weaning (1.3%) but substantial in terms of ADG (20%) and OM digestibility (19%) during the grazing period.

In terms of rumen fermentation, (Fig 1D–L), from 6 to 23 weeks of age animals increased their rumen pH (6.37 ± 0.26 vs 7.03 ± 0.19, *p* < 0.001) and ammonia concentration (5.89 ± 2.67 vs 7.69 ± 2.13 mg/dl, *p* < 0.001) but decreased acetate (64 ± 6 vs 60 ± 3%, *p* < 0.001) and lactate levels (15.0 ± 7.5 vs 5.0 ± 1.9 mM, *p* < 0.001) across treatments. The rearing system exerted different short‐ and long‐term effects on the rumen fermentation, because significant interactions (R × A) were observed. At weaning, NN lambs had higher volatile fatty acid (VFA) concentrations (95 ± 35 vs 67 ± 24 mM, interaction *p* < 0.001) and butyrate molar proportion (13.1 ± 4.6 vs 8.5 ± 3.3%, interaction *p* = 0.002), whereas artificially reared lambs had higher valerate levels (2.0 ± 0.9 vs 1.5 ± 0.5%, interaction *p* = 0.034). These differences in rumen fermentation across rearing systems were not observed at 23 weeks of age.

### *Microbial concentration*

**Figure 2:**
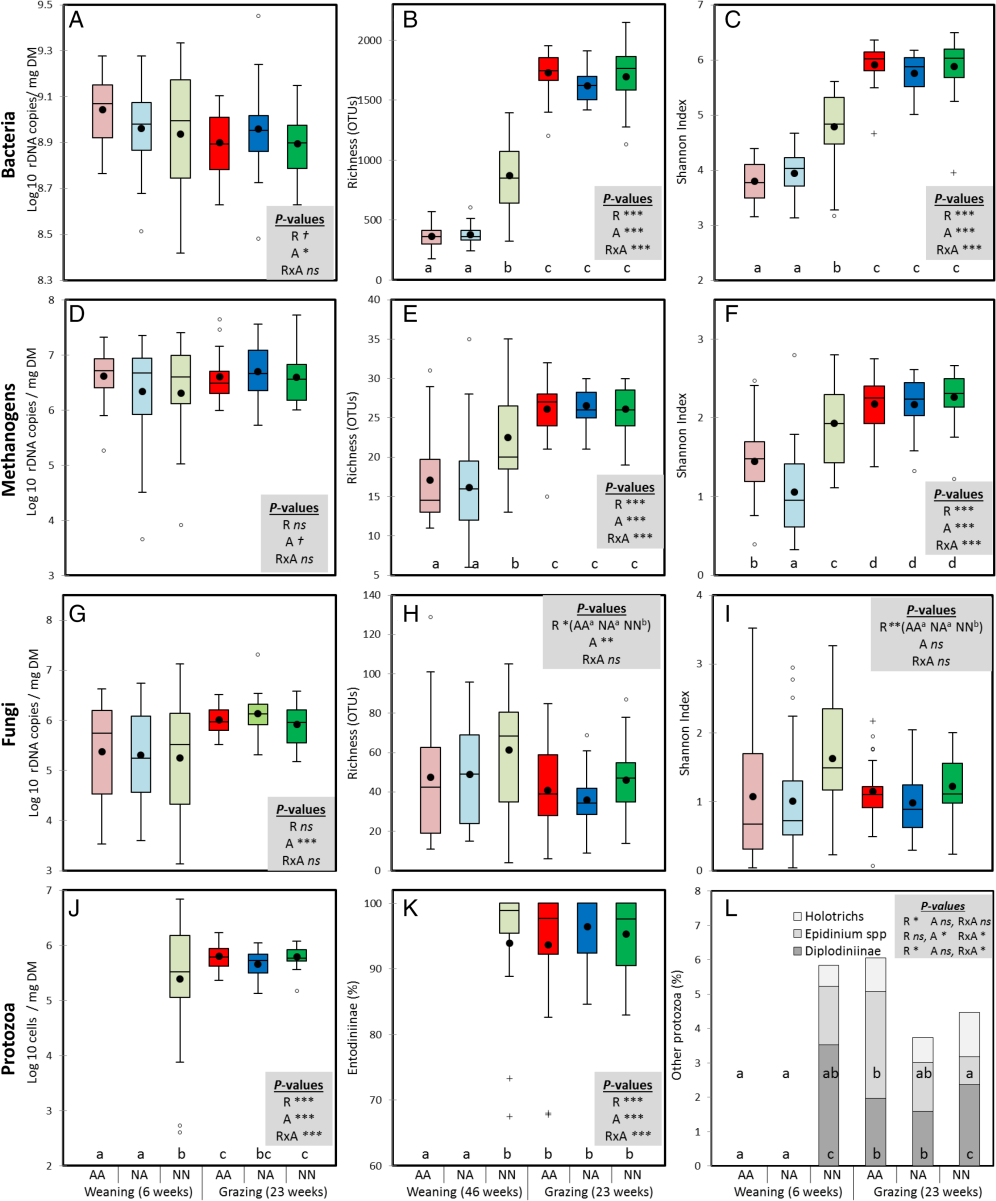
Boxplot indicating the short‐ (6 weeks of age) and long‐term effects (23 weeks) of the rearing system on the concentration and diversity of the main microbial groups in the rumen of lambs. Treatments: AA, colostrum alternative and artificial milk feeding, NA, ewe colostrum and artificial milk feeding; NN, natural rearing. ***p < 0.001; **p < 0.01; *p < 0.05; †*p* < 0.1; ns, not significant for the effects of rearing (R), age (A) and interaction (R × A). Boxes without a common letter differ based on a significant interaction. [Color figure can be viewed at http://wileyonlinelibrary.com]

Quantitative PCR showed no differences across rearing systems on the rumen concentration of total bacteria, methanogens and anaerobic fungi (Fig 2A, D and G). However, all lambs experienced an increase in the rumen concentration of methanogens (6.43 ± 0.83 vs 6.67 ± 0.49 log/mg DM, *p* = 0.057) and anaerobic fungi (5.32 ± 1.01 vs 6.04 ± 0.38 log/mg DM, *p* < 0.001), as well as a decrease in the bacterial concentration (8.98 ± 0.20 vs 8.92 ± 0.49 log/mg DM, *p* = 0.047), from week 6 to week 23 of age.

Examination of the rumen protozoal community by optical microscopy (Fig 2J–L) revealed that artificially reared lambs (AA and NA) had no rumen protozoa at weaning. On the contrary, natural rearing promoted an early colonization of the rumen by a diverse and variable protozoal population. This protozoal population grew in size during the grazing period leading to high and more constant concentration of rumen ciliates at 23 weeks of age across all treatments (interaction, *p* < 0.001). Protozoal community was always dominated by the subfamily Entodiniinae (94.9 ± 0.5%) and at 23 weeks of age NN had higher proportion of Diplodiniinae (2.4 ± 4.5 vs 1.8 ± 2.9%, interaction *p* = 0.050) and lower proportion of *Epidinium spp*. (0.8 ± 2.2 vs 2.3 ± 5.3%, interaction, *p* = 0.023) than artificially reared lambs.

### *Microbial diversity*

Next‐generation sequencing (NGS) produced 10.7, 0.8 and 5.3 million high‐quality sequences and samples were normalized at 12 500 ± 127, 1098 ± 2 and 8901 ± 4 reads per sample for bacteria, methanogens and fungi, respectively. After normalization, samples retained reasonable Good's coverage for bacterial (>94%), methanogens (>70%) and fungal (>63%) samples, with similar values to previous studies (Belanche *et al*., 2016a,b). A significant interaction was noted in terms of bacterial and methanogens diversity (Fig 2B, C, E and F). As a result at weaning, NN lambs had higher bacterial and methanogen diversity (871 ± 367 vs 367 ± 88 bacterial OTUs and 22 ± 6 vs 16 ± 6 methanogen OTUs) and Shannon index than artificially reared lambs. However, during the post‐weaning period, the bacterial and methanogen communities increased in diversity reaching similar and low variable values across treatments (1679 ± 195 and 26 ± 3 OTUs for bacteria and methanogens respectively). The fungal diversity (Fig 2H and I) decreased with the age of the lambs for all rearing systems (from 53 ± 29 to 41 ± 17 OTUs, *p* = 0.002); however, NN lambs had consistently higher fungal diversity values than artificially reared lambs independently of the age of the animals (54 ± 24 vs 43 ± 23 OTUs and 1.43 ± 0.60 vs 1.05 ± 0.71 Shannon index, respectively, *p* < 0.05).

### *Microbial communities and taxonomy*

**Figure 3:**
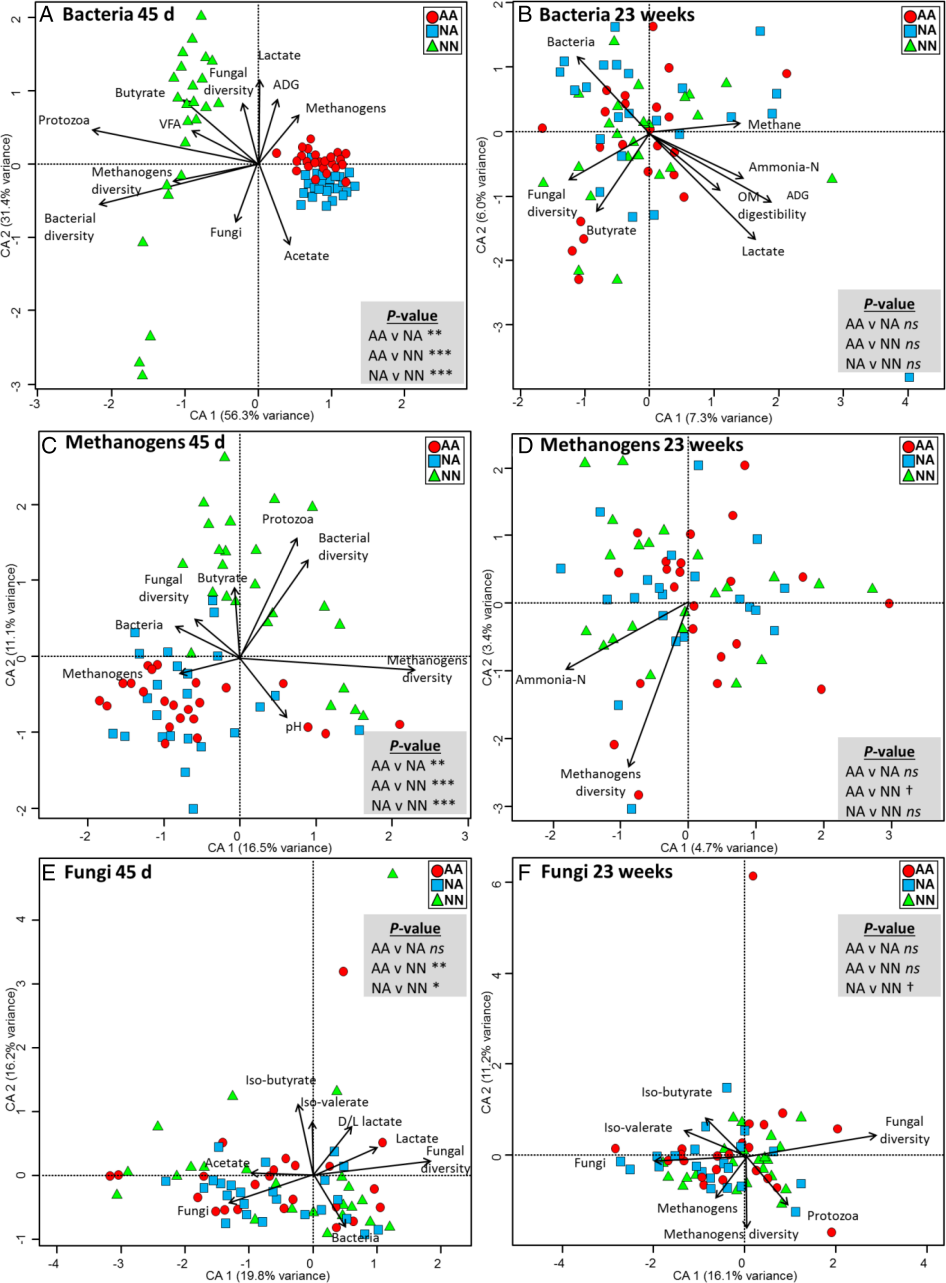
Canonical correspondence analysis describing the short‐ and long‐term effects of the rearing system on the relationships between the structure of the bacterial (A, B), methanogens (C, D) and fungal (E, F) communities with the rumen function in lambs. Only significant variables are shown (*p* < 0.05). Treatments: AA, colostrum alternative and artificial milk feeding, NA, ewe colostrum and artificial milk feeding; NN, natural rearing. Pairwise PERMANOVA values are provided in grey boxes, ***p < 0.001; **p < 0.01; *p < 0.05; †*p* < 0.1; ns, not significant. [Color figure can be viewed at http://wileyonlinelibrary.com]

Permutational analysis of variance (PERMANOVA, Table 1) and canonical correspondence analysis (CCA, Fig. 3) of the bacterial OTUs showed that the structure of the bacterial community was mainly determined by the age of the animal (explaining 25.5% of the variance), followed by the interaction R × A (12.0%) and the rearing system (7.82%). As a result of this interaction, the three rearing systems promoted large differences in the bacterial community structure at weaning, being particularly evident between NN and artificially reared lambs (Figs 3A and Supporting Information S1). CCA of the rumen bacterial community at weaning captured 87.7% of the variance and showed that this community in NN lambs was positively correlated with VFA, butyrate, protozoal concentration and diversity of the bacterial, methanogens and fungal communities suggesting a high microbial activity (Fig. 3A). During the grazing period, these differences between treatments disappeared (CCA explained 13.3% of the variance), but the bacterial community structure was still correlated with parameters of interest such as OM digestibility, body weight gain or methane production (Fig. 2B).

**Table 1:** PERMANOVA illustrating the short‐ (6 weeks of age) and long‐term effects (23 weeks of age) of early life management on the structure of the bacterial, methanogens and fungal communities in the rumen based on the Bray–Curtis dissimilarity.

	Rearing	Age	R × A
*Bacteria*			
Variance (%)	7.82	25.5	12
Pseudo‐F	9.20	60.0	9.08
*P*‐value	<0.001	<0.001	<0.001
*Methanogens*			
Variance (%)	6.23	27.8	16.5
Pseudo‐F	7.92	70.6	7.09
*P*‐value	<0.001	<0.001	<0.001
*Fungi*			
Variance (%)	2.44	18.1	18.7
Pseudo‐F	2.22	32.9	1.69
*P*‐value	0.005	<0.001	0.029

**Figure 4:**
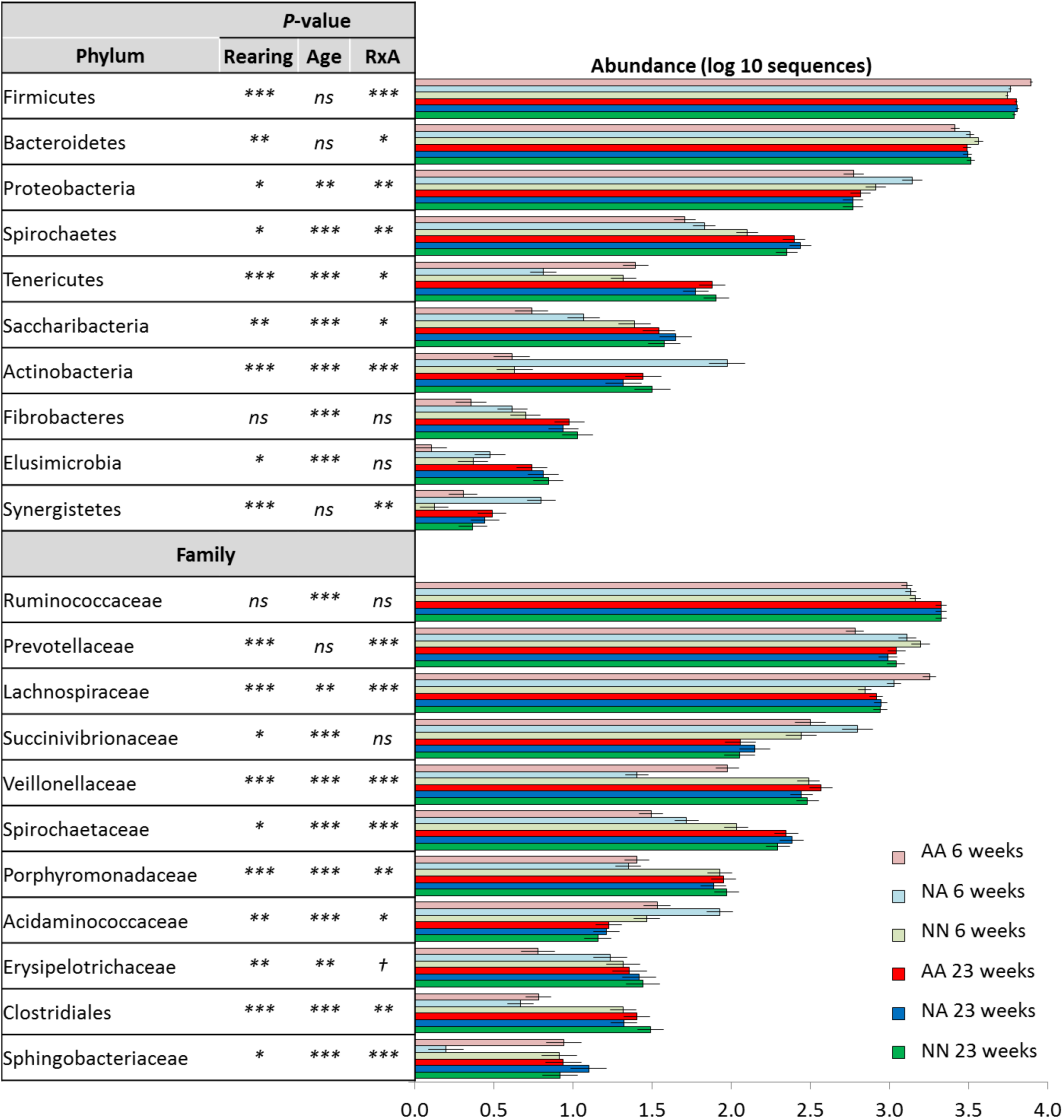
Short‐ (6 weeks old) and long‐term effects (23 weeks) of the rearing system on the relative abundance of the bacterial taxa in the rumen of lambs. Treatments: AA, colostrum alternative and artificial milk feeding, NA, ewe colostrum and artificial milk feeding; NN, natural rearing. Error bars show standard error of the mean. ***p < 0.001; **p < 0.01; *p < 0.05; †*p* < 0.1; ns, not significant. [Color figure can be viewed at http://wileyonlinelibrary.com]

In terms of bacterial community structure, a significant interaction (R × A) was noted for most bacterial taxa at phylum and family levels (Figs 4 and Supporting Information S2). At weaning, NN lambs showed the highest abundance of the phyla Bacteroidetes (mainly *Prevotella*), Spirochaetes and the genera *Flavonifractor* and *Syntrophococcus*, as well as the lowest abundance of phylum Firmicutes (*Ruminococcus* and *Megasphaera*). High ruminal levels of Spirochaetes, *Prevotella*, *Flavonifractor* and *Syntrophococcus* in NN lambs seemed to indicate bacterial community development since the abundance of these taxa increased as the rumen matured from 6 to 23 weeks of age across treatments. On the contrary, AA lambs at weaning had the lowest abundance of Proteobacteria and Spirochaetes suggesting a low rumen bacterial development, whereas NA lambs had an intermediate situation with high levels of *Butyrivibrio*, *Pseudobutyrivibrio* and *Ruminococcus*. As a result of the higher bacterial maturity in NN at weaning, the heatmap showed that the shift in the bacterial taxon distribution driven by the age was less obvious for NN than for artificially reared lambs (Supporting Information Fig. S1). However, a late bacterial community development in artificially reared lambs allowed them to catch up during the grazing period resulting in a lack of differences among rearing systems in terms of bacterial community structure and taxon distribution at the end of the grazing period (Figs. 3B and 4).

PERMANOVA (Table 1) showed that the methanogen community structure was also mainly determined by the age of the animal (27.8% of the variance), followed by the interaction R × A (16.5%) and the rearing system (6.23%). Pairwise analysis showed that the rearing system had a strong effect on the structure of this community at weaning, with the greatest differences observed between lambs receiving natural versus artificial milk feeding (Fig. 3C and Supporting Information Fig. S3). CCA of the methanogens at weaning captured 27.6% of the variance and showed that community structure in NN lambs was positively correlated with the protozoal and butyrate concentrations and with bacterial and methanogen diversities. During the grazing period, the methanogen community structure was correlated with the level of ammonia (Fig. 3D), but differences between rearing systems disappeared, possibly as a result of the low variance captured in the CCA (8.1%).

**Figure 5:**
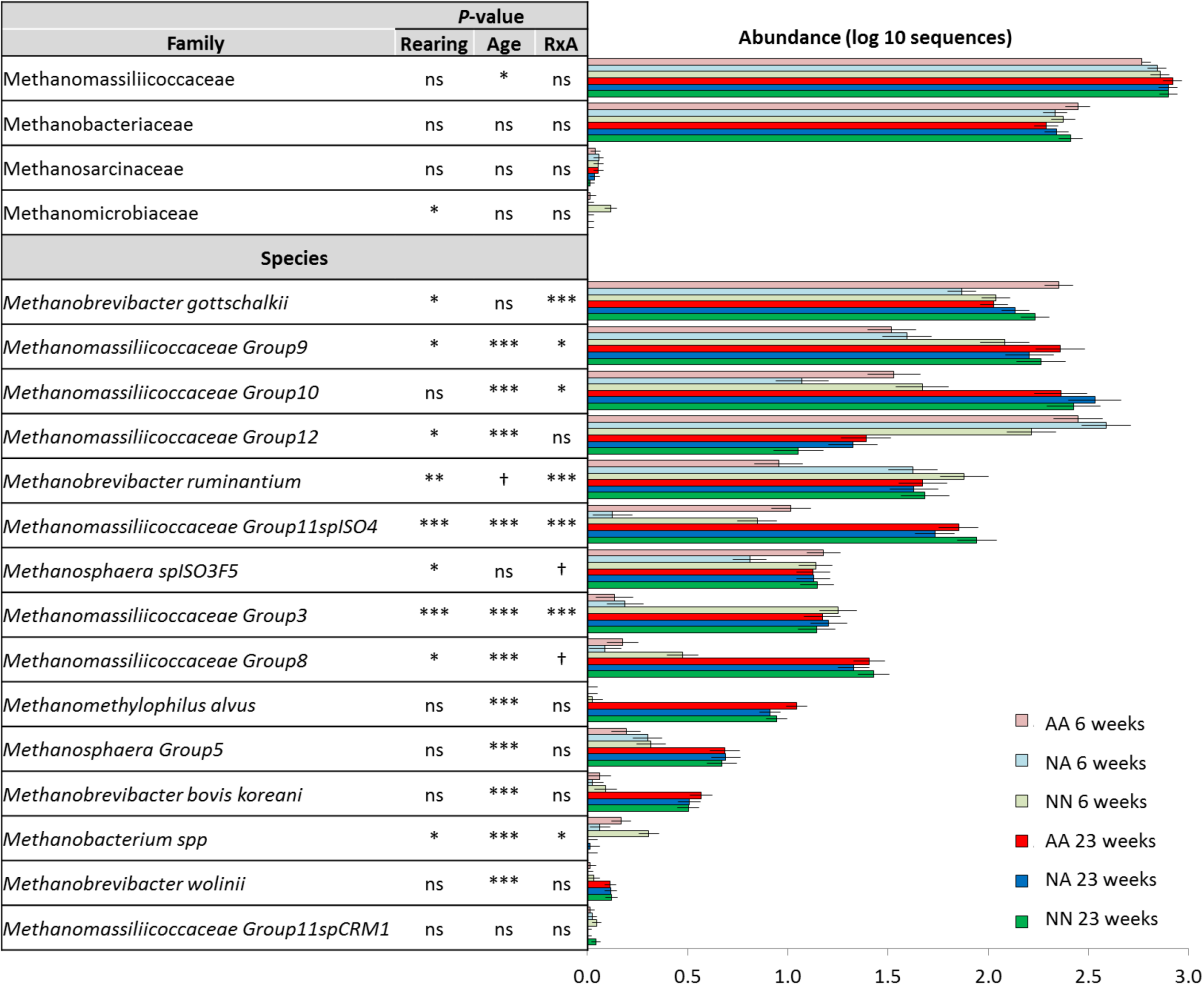
Short‐ (6 weeks old) and long‐term effects (23 weeks) of the rearing system on the relative abundance of the methanogen taxa in the rumen of lambs. Treatments: AA, colostrum alternative and artificial milk feeding, NA, ewe colostrum and artificial milk feeding; NN, natural rearing. Error bars show standard error of the mean. Error bars show standard error of the mean. ***p < 0.001; **p < 0.01; *p < 0.05; †*p* < 0.1; ns, not significant. [Color figure can be viewed at http://wileyonlinelibrary.com]

In terms of methanogen taxon distribution (Fig. 5), most taxa showed a significant interaction R × A. At weaning, higher levels of *Methanobrevibacter ruminantium* and lower levels of *Methanobrevibacter gottschalkii* and Methanomassiliicoccacea group 11 were noted in NA than in AA lambs, whereas NN lambs had higher abundance of *M. ruminantium*, *Methanobacterium* and Methanomassiliicoccaceae groups 3 and 9 than artificially reared lambs. From the heatmap (Supporting Information Fig. S3), it is clear that the shift in the methanogens community driven by age was less evident for NN lambs than for artificially reared lambs, suggesting a delayed development of the methanogens community for the latter group. Although some of these differences in community structure and taxon abundance disappeared during the grazing period, a small residual effect persisted at 23 weeks of age. As a result, NN lambs had higher rumen concentration of *Methanosphaera* and Methanomassiliicoccacea group 8 along with lower levels of group 12 across ages. High levels of *M. ruminantium* together with a substitution of Methanomassiliicoccaceae group 12 by other groups (mainly 3, 8, 9, 10 and 11) appeared to act as indicators of the methanogens community development because these changes also occurred as an adaptation to grazing diet across treatments.

The study of the fungal community structure by PERMANOVA (Table 1) showed that the impacts of lamb's age and the interaction RxA were the main drivers, explaining 18% of the variance, while the rearing system (2.4%) had limited impact on fungal community structure. Pairwise analysis showed differences in the fungal community structure at weaning between NN and artificial reared lambs but not between AA and NA lambs (Fig. 3E and Supporting Information S4). CCA of the fungal community at weaning captured 36.0% of the variance and showed that fungal community structure was correlated with lactate, acetate, iso‐valerate and iso‐butyrate levels. CCA of fungal community structure during the grazing period captured 27.3% of the variance and showed that the structure of this community was correlated with the concentration of protozoa, fungi and methanogens, despite no substantial differences were noted across treatments (Fig. 2F).

**Figure 6:**
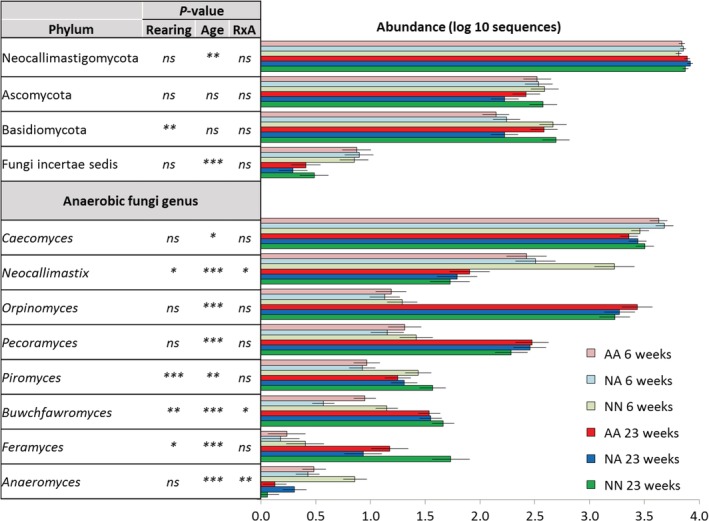
Short‐ (6 weeks old) and long‐term effects (23 weeks) of the rearing system on the relative abundance of the fungal taxa in the rumen of lambs. Treatments: AA, colostrum alternative and artificial milk feeding, NA, ewe colostrum and artificial milk feeding; NN, natural rearing. Error bars show standard error of the mean. ***p < 0.001; **p < 0.01; *p < 0.05; †*p* < 0.1; ns, not significant. [Color figure can be viewed at http://wileyonlinelibrary.com]

Regarding fungal taxon distribution, anaerobic fungi (phylum Neocallimastigomycota) represented the great majority of the fungi present (85.1 ± 17.5%), but a smaller proportion of aerobic fungi that were likely ingested with the feed, including yeasts (3.7 ± 8.4%), plant‐pathogens (3.7 ± 4.5%), saprophytes (3.3 ± 5.2%) and unclassified fungi (4.2 ± 4.3%) were also detected. The use of generic fungal primers has been validated for rumen studies (Edwards *et al*., 2017) and although most fungal species entering the rumen with the feed are obligate aerobes and thus considered to be transient and non‐functional (Bauchop, 1979), some (e.g. yeast) can have modulatory effects on the rumen function (Newbold *et al*., 1996). Natural rearing promoted an early development of the anaerobic rumen fungal community visible at weaning and consisting of increased abundance of key anaerobic fungi such as *Neocallimastix*, *Buwchfawromyces* and *Anaeromyces*, but those differences disappeared during the grazing period (interaction, *p* < 0.05). Moreover, NN lambs had higher levels of *Piromyces* (*p* = 0.007) and *Feramyces* (*p* < 0.001) than artificially reared lambs across sampling times. The abundances of *Piromyces* and *Feramyces* (together with *Buwchfawromyces*, *Orpinomyces* and *Perocamyces*) in the rumen may be indicators of the rumen fungal development because their abundance increased with the age of the lambs (Fig. 6).

## Microbial correlations

A number of correlations were found between the rumen microbes and fermentation parameters at weaning (Table 2). For example, rumen ammonia concentration was positively correlated with the abundance of Prevotellaceae (*ρ* = 0.48) and Neocallimastiigomycota (*ρ* = 0.40) but negatively correlated with fungal richness (*ρ* = −0.44). Rumen protozoal concentration (and more specifically Entodiniinae) was positively correlated with total VFA concentration (*ρ* = 0.45) and butyrate molar proportion (*ρ* = 0.51). Moreover, the ADG during the post‐weaning period (from 6 to 13 weeks of age) was positively correlated with the abundance of several anaerobic fungi genera, including *Neocallimastix* (*ρ* = 0.55), *Piromyces* (*ρ* = 0.53), *Buwchfawromyces* (*ρ* = 0.42) and *Pecoramyces* (*ρ* = 0.40). However, at 23 weeks of age, fewer correlations were observed between rumen microbes and productive outcomes. At this age, abundance of *Methanosphaera* was positively correlated with ammonia concentration (*ρ* = 0.53) and ADG during the grazing period (*ρ* = 0.40), whereas Prevotellaceae negatively correlated with both parameters (*ρ* = −0.42 and −0.42, respectively).

**Table 2:** Correlations between rumen microbes and rumen fermentation and animal performance data. [Color table can be viewed at http://wileyonlinelibrary.com]

	Weaning (6 weeks)								Grazing (23 weeks)		
Parameters^a^	pH	Ammonia	VFA	Acetate	Propionate	Butyrate	Lactate	ADG‐pw	Ammonia	Propionate	ADG‐f
Bacterial community											
Family Prevotellaceae		0.48							−0.42		−0.42
*Hydrogenoanaerobacterium*		0.42									
*Paraprevotella*	−0.42		0.43								
*Pseudoflavonifractor*		0.47				−0.55					
*Sphaerochaeta*	−0.40					0.40					
Methanogens community											
Family Methanobacteriaceae									0.41		
Family Methanomassiliicoccaceae									−0.41		
*Methanobrevibacter ruminantium*									0.47		
*Methanosphaera*									0.53		0.40
Fungal community											
Concentration				0.52							
Rinchness				−0.54			0.41				
Shannon index		−0.44		−0.52			0.40				
Phylum Ascomycota				−0.55	0.43		0.51	0.55			
Phylum Basidiomycota		−0.46		−0.57	0.41						
Phylum Neocallimastigomycota		0.40		0.57	−0.44		−0.43				
*Buwchfawromyces*								0.42			
*Caecomyces*				0.44							
*Neocallimastix*								0.55			
*Orpinomyces*		−0.40								−0.46	
*Pecoramyces*								0.40			
*Piromyces*								0.53			
Yeast				−0.47			0.48				
Protozoal community											
Concentration			0.43			0.49					
Subfamily Entodiniinae			0.45			0.51					

a Parameters: pH, ammonia‐N (mg/dl), total volatile fatty acids (mM), acetate (%), propionate (%), butyrate (%), lactate (mM) and average daily gain (kg/d) during the post‐weaning (ADG‐pw, 6–13 weeks) and finishing periods (ADG‐f, 13–23 weeks of age). Microbial data were log transformed and only Spearman's correlations coefficients ≥0.40 (green) or ≤ −0.4 (red) and *p* < 0.001 were shown (*n* = 72).

### *Core rumen microbiota*

**Figure 7:**
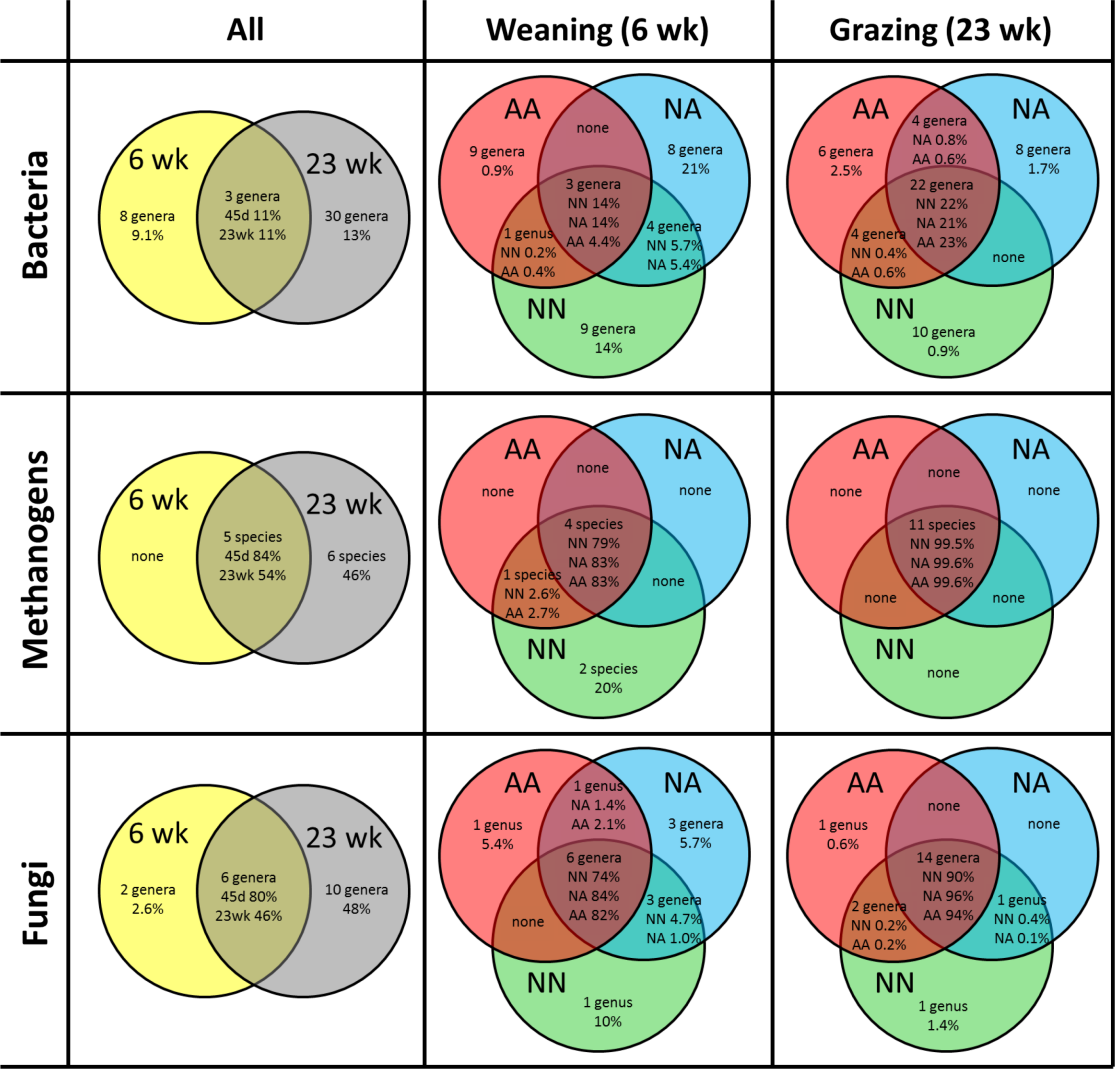
Venn diagrams describing the short‐ (6 weeks of age) and long‐term (23 weeks) effects of the rearing system on the core microbial communities in the rumen of lambs. The number of taxonomical entities and their perceptual contribution to the community is described. The core community is defined as those microbial taxa present in more than 95% of the individuals. Treatments: AA, colostrum alternative and artificial milk feeding, NA, ewe colostrum and artificial milk feeding; NN, natural rearing. [Color figure can be viewed at http://wileyonlinelibrary.com]

The core bacterial community across all samples comprised three genera (*Prevotella*, *Ruminococcus* and Lachnospiraceae) and represented approximately 11% of the total community (Fig. 7). Venn diagrams showed that for each treatment, there was an associated core community which increased with the age of the lambs. NN lambs shared a greater core bacterial community (+3 genera) in comparison to artificially reared lambs at weaning but not at grazing. The core methanogenic community across all samples comprised five species (*M. gottschalkii*, *M. ruminantium*, *Methanosphaera* ISO3F5 and Methanomassiliicoccaceae group 9 and 12), which represented 84% and 54% of the methanogen community at 6 and 23 weeks of age respectively. This core community also increased with the age of the lambs and was greater in NN lambs than in artificially reared lambs (+3 species) at weaning but not at grazing. The overall core fungal community was represented by six fungal genera: the anaerobic fungi *Caecomyces*, *Neocallimastix* and, *Piromyces*, as well as the aerobic *Phaeosphaeria*, *Alternaria* and *Filobasidium*, which represented 80% and 46% of the fungal community at 6 and 23 weeks of age respectively. This core fungal community increased with the age of the animals and the rearing system had minor effects at weaning but not at 23 weeks of age when NN lambs had a more diverse core community (+2 genera) than artificially reared lambs.

## Discussion

### *Effect of postnatal feeding*

This study investigated two widely used post‐natal management regimes based on (i) maximizing maternal colostrum intake (NN and NA lambs) and (ii) colostrum alternative supplementation (AA lambs). In newborn ruminants, the initial microbial community in the developing rumen is acquired from the surrounding community, including the vagina during delivery, skin during suckling or grooming, rearing material, colostrum and milk (Wang *et al*., 2016; Yeoman *et al*., 2018), with bacterial concentrations reaching 10^9^ cells ml^−1^ by the second day after birth (Fonty *et al*., 1989). Thus, colostrum is particularly relevant because it provides, along with immunological factors, the earliest form of nutrition both to the newborn lamb and to the developing rumen microbiota, as well as autochthonous microbes (Yeoman *et al*., 2018). In a companion paper, we demonstrated that lambs fed colostrum alternative tended to have lower plasma IgG concentrations at 24 h after birth and higher diarrheal events during the milk‐feeding period in comparison to NA lambs (Belanche *et al*., 2019a). In this article, we expand these findings to show that the type of colostrum can also have an impact on the rumen microbiota, despite all siblings were exposed to the same post‐natal environment during birth.

Three steps have been defined in the rumen microbial colonization: during the first 2 days after birth, the rumen is populated by pioneer colonizers (Skillman *et al*., 2004). Among them Proteobacteria, which is highly abundant in the colostrum (Yeoman *et al*., 2018), is the most abundant phylum (90%) (Jiao *et al*., 2015) and contributes to decreasing the oxygen partial pressure (Rey *et al*., 2014), which in turn allows strictly anaerobic microbes to colonize the rumen in a second stage from day 2 to 14. In a third stage (14 days to weaning), the rumen microbiota grows in complexity, becoming dominated by Bacteroidetes and Firmicutes as a result of a gradual increased in solid feed intake (Jami *et al*., 2013).

The current study showed that at weaning lambs fed maternal colostrum had increased levels of early rumen colonizers involved in starch degradation such as Actinobacteria and Proteobacteria (mostly *Ruminobacter*) or involved in H_2_ capture (*M. ruminantium*), which together with higher VFA concentrations (+29%) indicated greater rumen microbial fermentation in comparison to AA lambs. These results suggested that natural colostrum, relative to colostrum alternative, can favour early rumen microbial colonization leading to improvements of rumen function detectable at weaning. These findings are consistent with accumulating evidence showing that quantity and quality of colostrum intake determines to a great extent the future productive outcomes in dairy cows (DeNise *et al*., 1989; Faber *et al*., 2005).

### *Short‐term effects of the rearing system*

The aim of this study was to compare not only the effect of the two milk types (maternal vs milk replacer), but the whole rearing system, and this also accounts for the presence/absence of the dam. Our experiment showed that NN lambs at weaning, in comparison to artificially reared lambs, had a microbiologically more mature rumen, likely as a result of two factors: (i) a higher solid feed intake resulting from a feeding behaviour learned from the dams (Vieira *et al*., 2012) or from a limited milk yield during the late lactation (Meale *et al*., 2016) and (ii) a role of dams as microbial ‘inoculators’ of the lambs (Abecia *et al*., 2014a,b). In our study, the presence of milk‐autochthonous bacteria (Quigley *et al*., 2013) in the rumen was negligible, suggesting a minor role of the ewe's milk as a source of microbial inoculum (Skillman *et al*., 2004).

A recent study showed that vaginal microbiota, followed by the skin of the udder and the colostrum play a role in populating the rumen of newborn calves with bacteria and archaea (Yeoman *et al*., 2018). Rumen protozoa are highly sensitive to oxygen and require direct contact between young and adult animals for an effective transmission, drinking water being the most likely mode of transfer of protozoal cells (Bird *et al*., 2010). Anaerobic fungi also are highly sensitive to oxygen, but their ability to form resistant spores allows them to retain viability in dung, soil and feed (McGranaghan *et al*., 1999). These microbial sources could explain the presence of a complex protozoal community in NN lambs along with greater bacterial, methanogen and fungal diversities, whereas artificially reared lambs lacked of rumen protozoa during the artificial milk‐feeding period. Similar microbiological features to those observed in NN lambs were reported when Merino lambs were inoculated early in life with rumen fluid from adult animals (De Barbieri *et al*., 2015), highlighting the role of the dams as microbial inoculator.

It is well established that the presence of rumen protozoa has modulatory effects on the rumen fermentation (Newbold *et al*., 2015) and here we conclude that rumen protozoa stimulated total VFA (+41%) and butyrate production (+52%) in NN lambs, because positive correlations were observed between protozoa levels and these fermentation products. Protozoa predatory activity may explain the low bacterial concentration in NN lambs (Belanche *et al*., 2012a,b). Moreover, the dilution of the microbiota by feed entering the rumen (mainly in NN lambs) could counterbalance microbial growth, leading to similar concentrations of methanogens and anaerobic fungi per gram of rumen digesta across treatments as previously reported (Belanche *et al*., 2012a,b).

Regarding the bacterial community, it was hypothesised that, similar to the human gut (Turnbaugh *et al*., 2009), there is a rumen ‘core’ microbiome that remains stable regardless of differences in diet. Petri *et al*. (2013) described a surprisingly stable bacterial ‘core’ microbiome in the rumen of adult cattle. Our study agrees with Petri *et al*., in that *Prevotella* and *Rumicococcus* are the main representatives of this core community, despite being less abundant in young animals. Moreover, the rearing system had an impact on the third stage of the rumen colonization process, which is dependent on the solid feed intake (Abecia *et al*., 2017). As a result, NN lambs experienced a shift in the bacterial community structure consisting of increased abundance of Bacteroidetes (mainly the proteolytic *Prevotella*), Spirochaetes (pectin degradation), *Syntrophococcus* (lignin demethylation), simple‐sugar degraders (*Lachnobacterium* and *Succiniclasticum*), butyrate producers (*Flavonifractor* and *Clostridiales*), as well as lower levels of Firmicutes. A similar high Bacteroidetes/Firmicutes ratio has been described in animals adapted to high concentrate diets (Fernando *et al*., 2010), indicating a mature rumen microbiota in NN lambs. In a previous study, we noted that the inoculation of defaunated sheep with protozoa also promoted an increase in the abundance of *Prevotella*, *Flavonifractor* and *Syntrophococcus* (de la Fuente *et al*., 2014), genera which here were identified as indicators of the rumen microbial development. These findings suggest that the presence of rumen protozoa in NN lambs may be a key driver in shaping the bacterial and methanogen community structure due to their symbiotic interactions (Belanche *et al*., 2014).

Methanogenic archaea are the only rumen microbes able to produce methane (Hook *et al*., 2010) and there has been significant research effort targeting this microbial community in order to decrease methane emissions (Hristov *et al*., 2015). Previous reports showed that methanogens (primarily Methanobacteriaceae) are early rumen colonizers, reaching maximal densities from 14 days after birth independently of feed intake (Skillman *et al*., 2004; Friedman *et al*., 2017). However, we observed a strong impact of the rearing system on the structure of the methanogen community and taxon distribution. As a result, NN lambs contained high levels of *M. ruminantium*, which has an obligate requirement for the coenzyme‐M methanogenesis factor, which is obtained exogenously by *M. ruminantium* from other methanogen species (Leahy *et al*., 2010). Thus, *M. ruminantium* has also been considered as an indicator of the rumen microbial maturity (Skillman *et al*., 2004; Abecia *et al*., 2014a,b).

Here, we demonstrated that the substitution of the Methanomassiliicoccaceae group 12 by other groups (mainly 3, 8, 9, 10 and 11) may also indicate fungal community development. Differences between these methanogens groups have been reported in terms of network correlations with lower connectivity for Methanomassiliicoccaceae group 12 than for their counterparts (Henderson *et al*., 2015). NN lambs also had high levels of *Methanobrevibacter* and *Methanomicrobium*, which are representatives of the active protozoal endo‐symbiotic community responsible of capturing H_2_ produced by protozoa (Sharp *et al*., 1998). However, no correlations were observed between methanogen taxa and rumen fermentation data at weaning suggesting a minor impact of this community on the rumen fermentation and productive outcomes.

All lambs had a complex fungal community at 6 weeks of age even in the absence of maternal contact, likely as a result of rumen colonization by fungal spores (McGranaghan *et al*., 1999). Moreover, the rearing system played a substantial role on shaping this community; NN lambs had a more mature fungal community in terms of diversity and abundance of important anaerobic monocentric fungi such as *Neocallimastix*, *Buwchfawromyces* and *Anaeromyces* in comparison to artificially reared lambs. The polycentric fungus *Anaeromyces* has a clear preference for glucose (Solomon *et al*., 2016), while *Neocallimastix* is a monocentric fibrolytic fungi involved in the early stages of feed colonization (Wood *et al*., 1986) and H_2_ production due to the presence of hydrogenosomes (Yarlett *et al*., 1986). The abundance of anaerobic fungi had a positive correlation with acetate and ammonia concentration suggesting a relevant role in the protein and fibre degradation (Edwards *et al*., 2008). The presence of a complex fungal community in the rumen had positive effects on the weaning process and the subsequent transition to a grass diet, as noted by the positive correlations observed between the ADG during the post‐weaning stage and the rumen concentration of key anaerobic fungi (*Neocallimastix*, *Pecoramyces*, *Piromyces* and *Buwchfawromyces*), which could be considered as indicators of the rumen fungal development. Similar short‐term positive effects on rumen function have been reported in lambs inoculated early in life with rumen fluid (De Barbieri *et al*., 2015) and in calves fed different diets (Dill‐McFarland *et al*., 2019), suggesting a plasticity in the rumen microbiome during the colonization process.

### *Long‐term effects of the rearing system*

Although the solid digesta‐associated microbiome was not investigated, the current study suggests that the age of the animals and the inherent diet change were the main drivers, which determined the structure of rumen microbiota and the fermentation patterns. During the grazing period, all animals were grouped on the same pasture with no supply of concentrates. This led to a decline in rumen VFA concentration (−46%), as well as to a homogenization of the rumen microbiome because a lower variability was observed across and within treatments. This microbial homogenization confirmed the importance of the microbial inter‐exchange, which occurs naturally between animals sharing the same flock, pasture and drinking water (Bird *et al*., 2010). This effect was particularly evident for the protozoal community because artificially reared lambs became faunated with a complex protozoal community during the grazing period.

The increase in diversity and core community of bacterial and methanogen populations as the lambs matured also indicated a progressive rumen colonization and subsequent consolidation of these microbial communities. In a recent study (Belanche *et al*., 2019b), we reported higher bacterial, methanogen and fungal diversity indices in the dams than in their 23 weeks‐old offspring, even when fed on the same pasture, suggesting that the rumen microbial colonization is a long‐lasting and continuous process (Jami *et al*., 2013).

The microbial response to the pasture diet involved increasing rumen abundances of Methanomassiliicoccaceae species along with fibrolytic bacteria (*Fibrobacter*, *Ruminococcus* and *Butyrivibrio*), consistent with a greater activity of bacterial cellulosomes (Berry, 2017). Anaerobic fungi are late rumen colonizers (Orpin and Joblin, 1997) and during the grazing period, this community grew in size but decreased in diversity because a few but highly abundant genera dominated the entire fungal population. In particular, *Orpinomyces* and *Pecoramyces* showed a large increase during the grazing period, possibly as a result of their longer life cycles, which makes growth possible in animals fed forage due to the longer rumen retention time (Dey *et al*., 2004). In contrast, *Anaeromyces* decreased in abundance due to the absence of starch in the grazing diet and potential predation of their zoospores by rumen protozoa (Lee *et al*., 2001).

The current study showed no residual effects of the rearing system on the bacterial community at 23 weeks of age. The methanogen community retained some long‐lasting effects derived from the rearing system and NN lambs had lower levels of Methanomassiliicoccaceae Group 12, considered an inefficient methylotrophic species (Liu and Whitman, 2008), and higher abundance of *M. gottschalkii*, which has been positively correlated with methane emissions (Tapio *et al*., 2017). These observations may suggest a more efficient interspecies H_2_ transfer and substrate oxidation in NN lambs (Liu and Whitman, 2008). However, no substantial correlations were found between levels of these methanogen taxa and productive outcomes under our experimental conditions. These findings suggest a minor (if any) feasibility of early life programming of the rumen prokaryotic community through the diet.

On the contrary, our study revealed for first time that rumen eukaryotic microbiota may be seeded early in early life because most of the positive effects induced by maternal rearing persisted during the grazing period. As a result, NN lambs retained higher levels of *Diplodiniinae* and holotrichs, both considered late rumen colonizers (Yáñez‐Ruiz *et al*., 2015), indicating an incomplete rumen faunation of artificially reared lambs during the grazing period. Similarly, at 23 weeks of age, NN lambs still retained signs of a high fungal community maturity such as greater fungal diversity, a more complex core microbiota and higher levels of key anaerobic fungi such as *Piromyces* and *Feramyces* than their artificially reared counterparts.

Although *Piromyces* has primarily been described as a cellulolytic fungus (de Souza, 2013), *Feramyces* is able to utilize a wider range of polysaccharides (Hanafy *et al*., 2018). Thus, the presence of these genera could facilitate the degradation of recalcitrant plant cell walls (Bauchop, 1979), penetrating the cuticle of ingested plant biomass and triggering the early colonization of the forage by other rumen microbes (Belanche *et al*., 2017). High fungal diversity and the presence of elevated levels of fibrolytic microbes (*Piromyces*, *Feramyces* and large Diplodiniinae protozoa) could be consistent with the observed increase in rumen VFA concentration (+4.3%), feed digestibility (+4.0%) and animal growth (+16%) in NN lambs during the grazing period. As a result, NN lambs had a higher final body weight (+7%), but only a slightly heavier carcass weight (+2.2%) than their artificially reared counterparts. This observation suggests that NN lambs had a heavier gastrointestinal tract derived from a larger rumen size or slower rumen transit time, which ultimately decreased the killing‐out percentage (Belanche *et al*., 2019a). However, no correlations were observed between these microbiological differences in the eukaryotic community and productive outcomes.

In conclusion, the current findings are in line with recent studies indicating that early life nutritional interventions can affect the initial rumen microbial establishment (De Barbieri *et al*., 2015) leading to positive short‐term effects (Zhong *et al*., 2014). Although some of these microbiological differences can persist later in life, post‐weaning factors have greater influence on rumen communities and productive outcomes (Dill‐McFarland *et al*., 2019). Therefore, it has been suggested that alterations of the microbiota for optimizing rumen function may be most effective during or immediately following the weaning transition (Dill‐McFarland *et al*., 2017).

## Experimental procedures

### *Animals and diets*

All animal procedures were conducted in accordance with the Home Office Scientific Procedures, Act 1986 and were authorized by the Aberystwyth University Ethics Committee (PLL 40/3653; PIL 40/9798). Twenty‐four pregnant ewes carrying triplets were selected based on the pregnancy scanning results. A total of 72 Aberdale‐Texel crossbreed lambs were born within an 8‐day period and one animal from each triplet set was allocated to each treatment taking into consideration the sex and initial body weight. As a result, all three groups had similar sex distribution (13 males, 11 females) and birth weights (3.8 ± 0.8 kg). Immediately after birth, one sibling (**AA**; Artificial–Artificial) was fed with 50 g of colostrum alternative divided in two equal doses (at 1 and 6 h after birth) and separated from its dam for artificial rearing with milk replacer. The other two siblings remained with their mother suckling ewe colostrum. Then, one of those lambs (**NA**; Natural–Artificial) was separated from its dam at 24 h after birth and artificially reared with milk replacer, while the third lamb (**NN**; Natural–Natural) remained with its mother suckling ewe milk until weaning at 6 weeks of age. Colostrum alternative and milk replacer were freshly prepared following the manufacturer instructions (Lamb Volostrum and Lamlac Instant, Volac, Lampeter, UK). During the milk‐feeding period, all experimental groups were kept in the same building but physically separated (1 m separation), with *ad libitum* access to creep feed (NuGro CCF, Aberystwyth, UK) and grass hay (Supporting Information Table S1). Animals were weaned at 6 weeks of age by abrupt weaning. In order to identify the persistence of effects of the rearing system on the rumen function and animal performance, all lambs were grouped together and grazed on the same ryegrass pasture (*Lolium perenne*) for the following 4 months. The ewes were also brought to the same pasture as the lambs during the last month until lambs reached 23 weeks of age.

### *Sampling and analyses*

In order to identify the short‐ and long‐term effects of the rearing system on the rumen microbiota, rumen sampling was performed at weaning (6 weeks) and at the end of the grazing period (23 weeks). Rumen content (ca. 50 ml) was withdrawn from each animal by orogastric intubation before the morning feeding (09:00 h). Rumen content was filtered through cheesecloth and solids were discarded given the small and variable proportion of solids in the rumen samples. Then, pH was measured and six subsamples were taken for microbial characterization (snap frozen in liquid N), VFA, ammonia, lactate and protozoal optical counting respectively (Belanche *et al*., 2016a,b). The last sample was incubated 24 h *in vitro* to determine gas and methane emissions (Belanche *et al*., 2015a,b). Rumen hydrogen production was stoichiometrically calculated (Marty & Demeyer, 1973). At 23 weeks of age, faecal samples were collected from each animal on two non‐consecutive days, which together with the pasture samples, were used to determine feed digestibility using acid insoluble ash as an internal marker (Thonney *et al*., 1979). For DNA extraction, freeze‐dried rumen samples were bead beaten and genomic DNA was extracted using the procedure described by Yu and Morrison (Yu and Morrison, 2004). Quantitative PCR was used to determine the absolute concentration bacteria, methanogens, anaerobic fungi and protozoa (Belanche *et al*., 2016a,b) using their respective primers (Supporting Information Table S2).

### *Next‐generation sequencing*

Rumen bacteria, methanogenic archaea and fungal communities were analysed using NGS DNA metabarcoding as previously described (Belanche *et al*., 2016a,b; Detheridge *et al*., 2016). For bacterial and methanogen sequencing, the amplification of the V1‐V2 and the V2‐V3 hypervariable regions of the 16S rRNA was performed, respectively, while for the fungi, the D1 variable region of the large (28S) subunit (LSU) of the rRNA locus was amplified. Primers used and PCR conditions are reported in the Supporting Information Table S2. Library preparation and sequencing were performed using an Ion Torrent system (Life Technologies, Paisley, UK). Sequences were trimmed to 300 bp length (200 bp for fungi) and Mothur software (version 1.37) was used to remove low‐quality sequences: maximum 10 homo‐polymers, Q15 average over 30 bp window and no mismatches with the primer/barcoding were allowed. Sequences were further screened for quality by discarding sequences with an expected error rate of 1 of greater using Uparse and chimera checking was performed using Uchime (Edgar and Flyvbjerg, 2015). Sequences were clustered into OTUs at 97% identity using Uclust and singletons were removed. The Ribosomal Database Project‐II Naïve Bayesian classifier (Version 11.1) was used for taxonomic classification against the curated bacterial (16S rRNA database) and fungal sequences (fungal LSU database) (Wang *et al*., 2007), while the 16S Rumen and Intestine Methanogen Database (RIM‐DB, Version 2.47) was used for methanogens (Seedorf *et al*., 2014). This approach allowed methanogens to be mostly classified at the species level, while bacteria and fungi were classified at the genus level. The number of reads per sample was normalized to the sample with the lowest number of reads to obtain similar sequencing depth. Raw sequences were deposited at the European Nucleotide Archive (accession numbers PRJEB33228 and PRJE34258).

### *Calculations and statistical analysis*

Statistical analyses were conducted using the Genstat software (18th Edition, VSN International, Hemel Hempstead, UK). Quantitative PCR data, protozoal cell counts and taxa abundances were tested for normality using the Shapiro–Wilk test and data were log‐transformed to achieve a normal distribution. Data were analysed based on a repeated measures mixed model (residual maximum likelihood) as follows:

$$Y_{\mathrm{ijk}}=\mu +R_{i}+T_{j}+{\left(R\times T\right)}_{\mathrm{ij}}+S_{k}+A{\left(S\right)}_{l}+e_{\text{ijkl}}$$

where *Y*_*ijk*_ is the dependent, continuous variable, *μ* is the overall population mean, *R*_*i*_ is the fixed effect of the rearing system (*i* = AA vs NA vs NN), *T*_*j*_ is the fixed effect of the sampling time (*j* = 6 vs 23 weeks), (*R × T*)_*ij*_ is the interaction term, *S*_*k*_ is the random effect of the sibling set considered as a block (*k* = 1–24), *A*_*l*_ is the random effect of the animal nested to the sibling set (*l* = 1–72) and *e*_*ijkl*_ is the residual error. When significant effects were detected, means were compared by Fisher's protected LSD test. Significant effects were declared at *p* < 0.05 and tendency to difference at *p* < 0.1. Animal sex and initial body weight were considered as block and covariate factors, respectively, for animal performance data but not for microbiological data, as no significations were observed.

The treatment effects on the rumen microbial communities were assessed based on the Bray–Curtis distance metrics using the unweighted pair group method with arithmetic mean function. Log‐transformed data were analysed by non‐parametric PERMANOVA using PRIMER‐6 software (PRIMER‐E, Plymouth, UK), as previously described (Belanche *et al*., 2017). A canonical correspondence analysis (CCA) was also conducted using R statistics (Vegan Package, Version 2.5) to explore the relationships between the structure of the rumen microbiota and the rumen fermentation. The significance of each variable was calculated after 999 random permutations. Taxa abundance was analysed using the Bonferroni statistical test to decrease the false discovery rate. Spearman correlations (*ρ*) were calculated to assess the relationships between the microbial taxa abundance and rumen fermentation and animal performance data. Strong correlations were defined as those with coefficients *ρ* ≥ 0.4 or ≤ −0.4 and *p* < 0.001. The core microbiota was calculated as those genera (or species) present across the vast majority of the individuals (>95%) of a given treatment (Turnbaugh *et al*., 2007).

## Supporting information


**Appendix S1:** Supporting Information
