# Supplementary material for: Maternal versus artificial rearing shapes the rumen microbiome having minor long‐term physiological implications
Source: Environ Microbiol. 2019 Oct 8;21(11):4360–77. doi: 10.1111/1462-2920.14801 (PMC6899609; doi:10.1111/1462-2920.14801)
Supplement: Supplementary file 1 — Appendix S1: Supporting Information [file EMI-21-4360-s001.docx]

**SUPPLEMENTAL MATERIAL**

**Maternal vs artificial rearing shapes the rumen microbiome having minor long-term physiological implications**

*Corresponding author: Alejandro Belanche a.belanche@csic.es

^
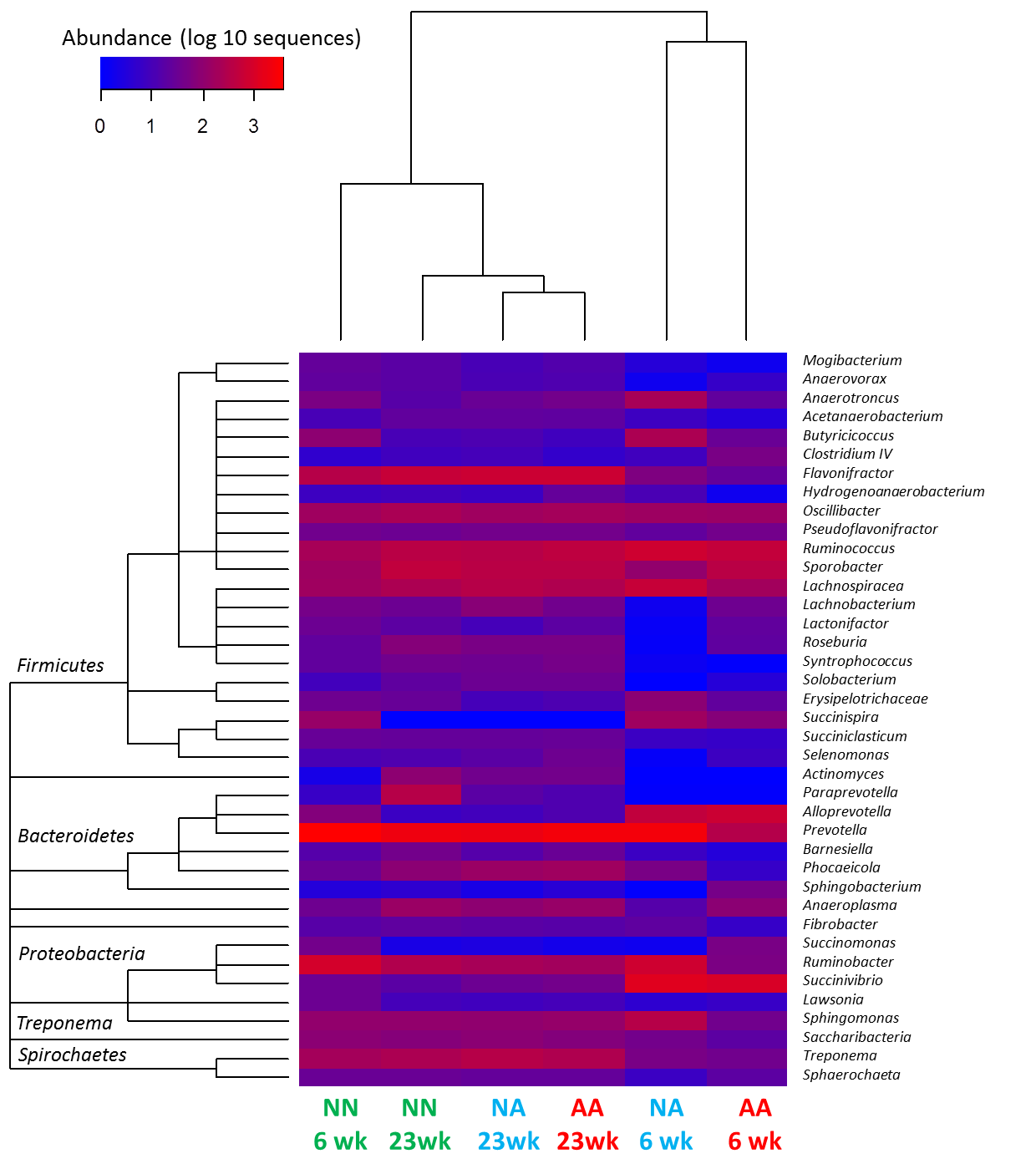
^

**Figure S1.** Heatmap describing the short- (6 weeks old) and long-term effects (23 weeks) of the rearing system on the structure of the bacterial community and the abundance of the main genera in the rumen (abundance above 0.05%). The dendrogram is based on the UPGMA clustering of Bray-Curtis distances. The total number of reads per sample was averaged per treatment, log10 transformed and minor genera discarded (67 genera representing 1.1% of the community). AA, colostrum alternative and artificial milk feeding, NA, ewe colostrum and artificial milk feeding; NN, natural rearing.

**
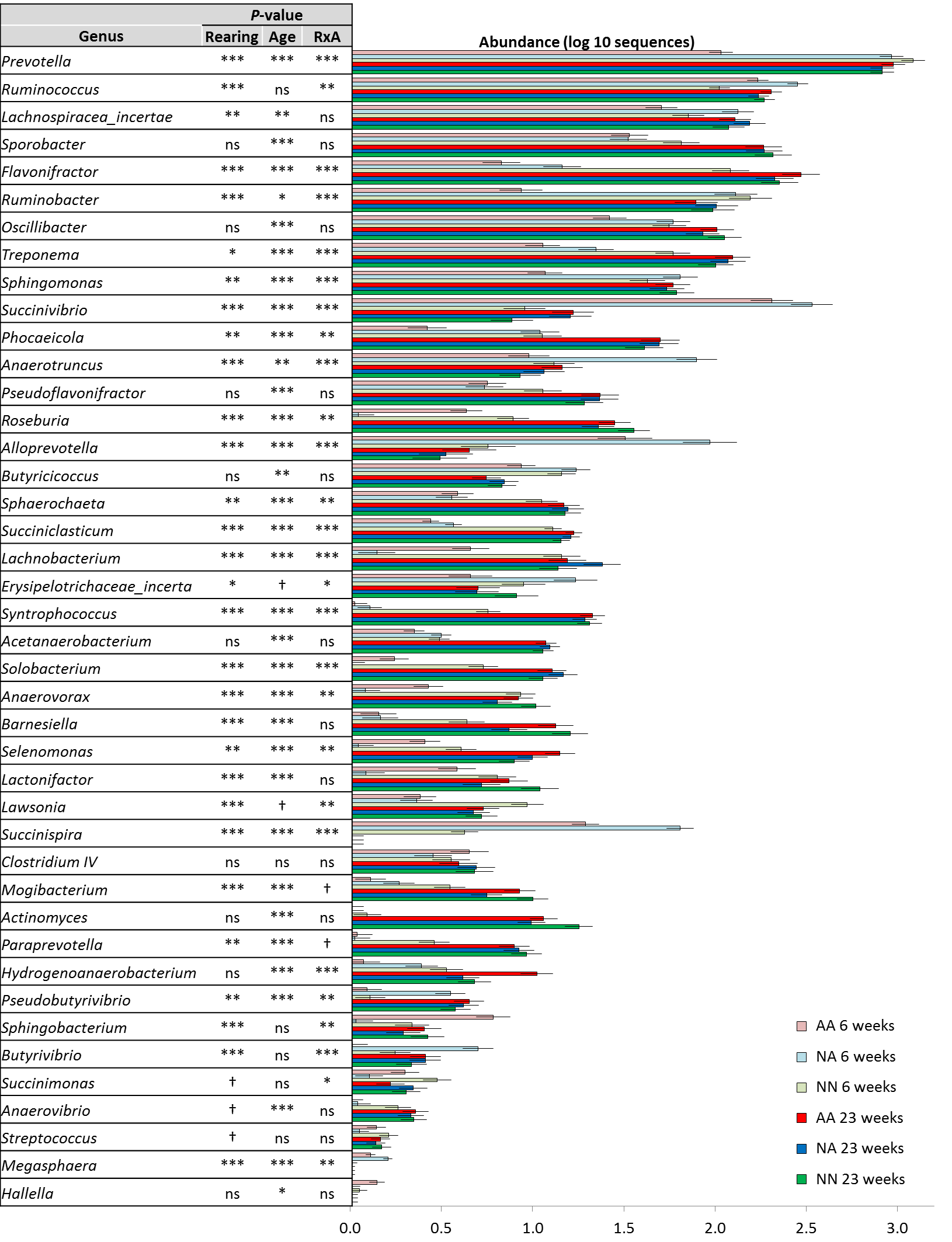
**

**Figure S2.** Short- (6 weeks old) and long-term effects (23 weeks) of the rearing system on the relative abundance of the bacterial genera in the rumen of lambs. Treatments: AA, colostrum alternative and artificial milk feeding, NA, ewe colostrum and artificial milk feeding; NN, natural rearing. Error bars show standard error of the mean. ****P*<0.001. ***P*<0.01. **P*<0.05. †*P*<0.1. *ns*, not significant.

**
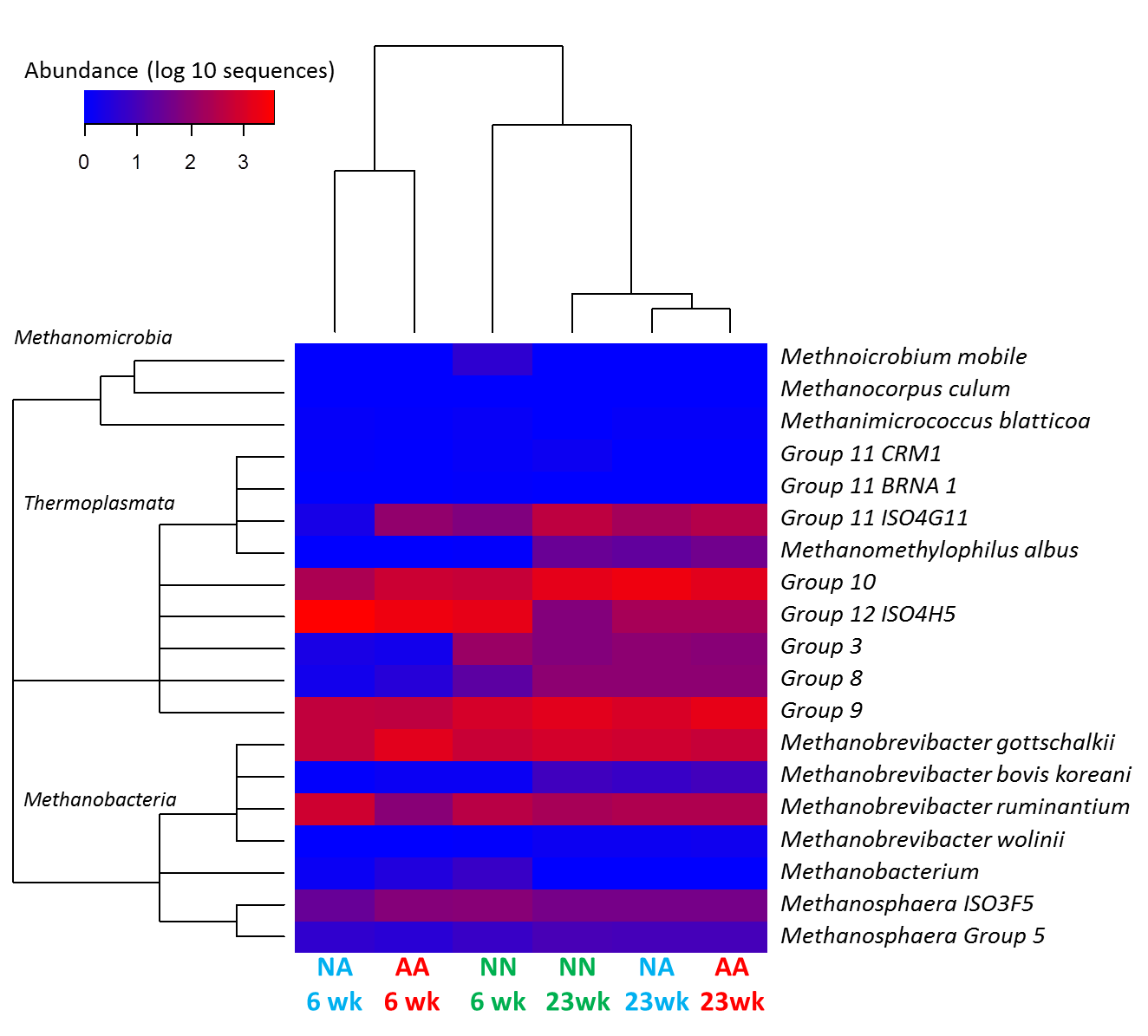
**

**Figure S3.** Heatmap describing the short- (6 weeks old) and long-term effects (23 weeks) of the rearing system on the structure of the methanogen community and species abundance in the rumen. The dendrogram is based on the UPGMA clustering of Bray-Curtis distances. The total number of reads per sample was averaged per treatment and log10 transformed. AA, colostrum alternative and artificial milk feeding, NA, ewe colostrum and artificial milk feeding; NN, natural rearing.


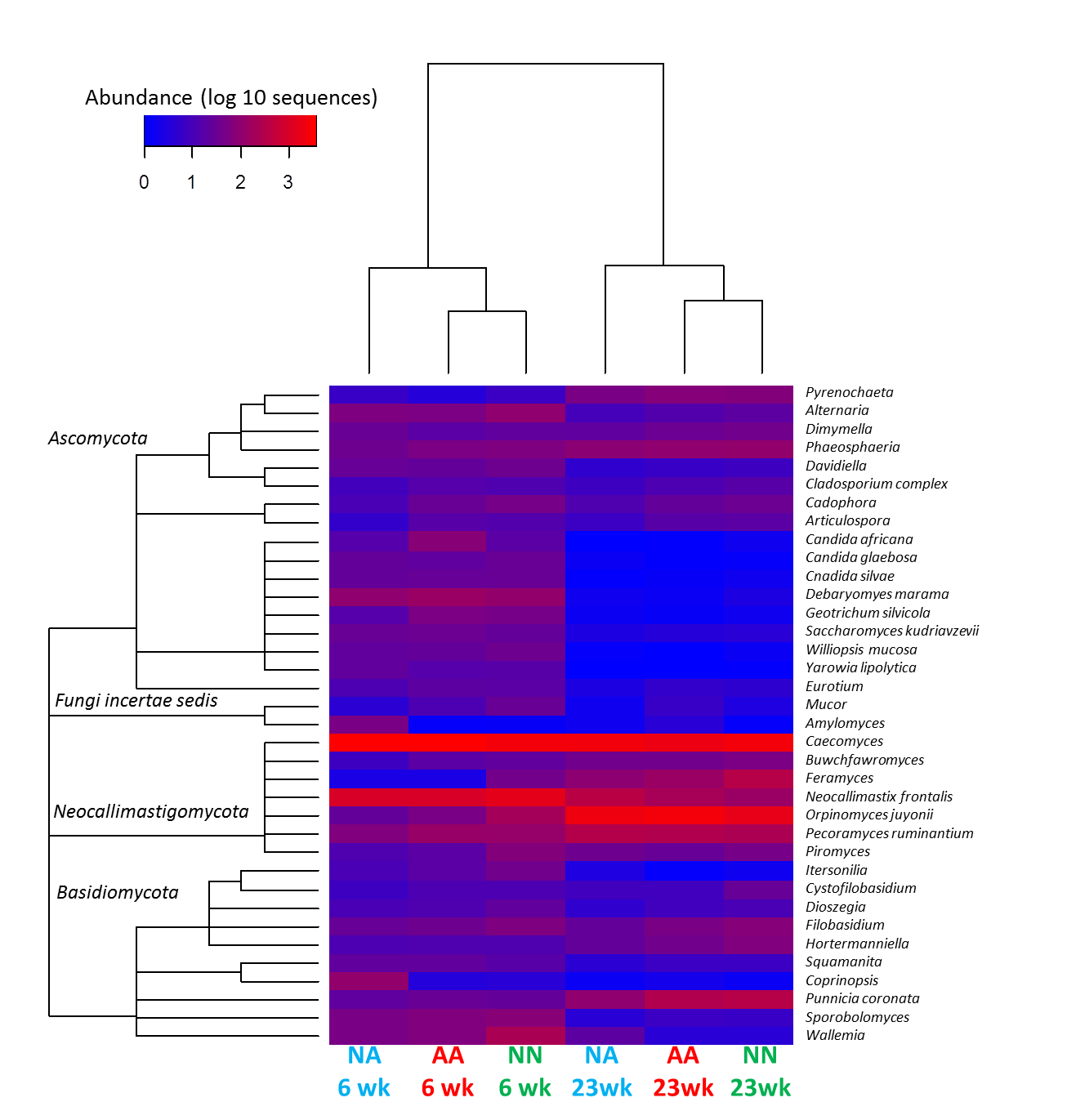


**Figure S4.** Heatmap describing the short- (6 weeks old) and long-term effects (23 weeks) of the rearing system on the structure of the fungal community and on the abundance of the main genera in the rumen (abundance above 0.1%). The dendrogram is based on the UPGMA clustering of Bray-Curtis distances. The total number of reads per sample was averaged per treatment, log10 transformed and minor genera discarded (57 genera representing 3.4% of the community). AA, colostrum alternative and artificial milk feeding, NA, ewe colostrum and artificial milk feeding; NN, natural rearing.

**Table S5.** Correlations between rumen microbes and fermentation and animal performance data

| **Parameter^1^** | **pH** | | **Ammonia** | | **VFA** | | **Acetate** | | **Propionate** | | **Butyrate** | | **Lactate** | | **ADG** | | **OMD** | **CH_4_** |
| --- | --- | --- | --- | --- | --- | --- | --- | --- | --- | --- | --- | --- | --- | --- | --- | --- | --- | --- |
| **Age (weeks)** | **6** | **23** | **6** | **23** | **6** | **23** | **6** | **23** | **6** | **23** | **6** | **23** | **6** | **23** | **6** | **23** | **6** | **23** |
| BACTERIAL COMUNITY |  |  |  |  |  |  |  |  |  |  |  |  |  |  |  |  |  |  |
| Richness |  |  |  |  |  |  |  |  |  | -0.35 |  |  |  |  |  |  |  | 0.30 |
| Phylum Actinobacteria |  |  |  |  |  |  |  |  |  | -0.33 |  |  |  |  |  |  |  | 0.37 |
| Phylum Bacteroidetes |  |  |  | 0.35 |  |  |  |  |  |  |  |  |  |  |  |  |  |  |
| Phylum Elusomicrobia |  |  |  |  |  | 0.32 |  |  |  |  |  |  |  |  |  |  |  |  |
| Phylum Fibrobacteres |  |  |  |  |  |  |  | -0.32 |  |  |  |  |  |  |  |  |  |  |
| Phylum Firmicutes |  |  |  |  |  |  |  |  |  | -0.31 |  |  |  |  |  |  |  |  |
| Family Acidaminococcaceae |  |  |  |  |  |  |  |  |  |  |  |  |  |  |  | -0.39 |  |  |
| Family Clostridiales |  |  |  |  |  |  |  |  |  |  | 0.38 |  |  |  |  |  |  |  |
| Family Prevotellaceae |  |  | 0.48 | -0.42 | -0.31 |  |  |  |  |  | -0.39 |  |  |  |  | -0.42 |  |  |
| Family Ruminoccocaceae |  |  |  |  |  |  |  |  |  |  |  |  |  |  | -0.33 |  |  |  |
| Family Succinivibrionaceae | -0.31 |  |  |  |  |  | -0.31 |  | 0.38 |  |  |  |  |  |  |  |  |  |
| Family Veillonellaceae |  |  |  |  |  | -0.37 |  |  |  |  |  |  |  |  |  |  |  |  |
| *Acetanaerobacterium* |  |  | 0.37 |  |  |  |  |  |  |  |  |  |  |  |  |  |  |  |
| *Alloprevotella* | 0.32 |  |  |  |  |  |  |  |  |  |  |  |  |  |  |  |  |  |
| *Anaerotruncus* |  |  | 0.37 |  |  |  |  |  |  |  |  |  |  |  |  |  |  |  |
| *Anaerotruncus* |  |  |  |  |  |  |  |  |  |  |  | 0.33 |  |  |  |  |  |  |
| *Anaerovorax* |  |  |  |  |  |  |  |  |  |  |  |  |  |  |  |  | 0.30 |  |
| *Barnesiella* | -0.33 |  |  |  |  |  |  |  |  |  |  |  | 0.32 |  |  |  |  | -0.33 |
| *Hydrogenoanaerobacterium* |  |  | 0.42 |  |  |  |  |  |  |  |  |  |  |  |  |  |  |  |
| *Lachnobacterium* |  |  | 0.32 |  |  |  |  |  |  |  | -0.34 |  |  | -0.36 |  |  |  |  |
| *Lawsonia* |  |  |  |  |  |  |  |  | 0.38 |  |  |  |  |  |  |  |  |  |
| *Oscillibacter* |  |  |  |  |  |  |  |  |  |  |  |  | -0.31 |  |  |  |  |  |
| *Paraprevotella* | -0.42 |  |  |  | 0.43 |  |  |  |  |  | 0.33 |  |  |  |  |  | -0.30 |  |
| *Pseudoflavonifractor* |  |  | 0.47 |  | -0.31 |  |  |  |  |  | -0.55 |  |  |  |  |  |  |  |
| *Roseburia* |  |  |  |  |  |  |  |  |  |  | 0.30 | 0.37 |  |  |  | -0.32 |  |  |
| *Ruminococcus* |  |  |  |  |  |  |  |  |  | 0.36 |  |  |  |  |  |  |  |  |
| *Selenomonas* |  |  |  |  |  |  |  |  | 0.34 |  |  |  |  |  |  |  |  |  |
| *Solobacterium* |  |  |  |  |  |  |  |  |  |  |  |  |  |  |  | -0.39 |  |  |
| *Sphaerochaeta* | -0.40 |  |  |  | 0.34 |  |  |  |  |  | 0.40 |  |  |  | 0.30 |  |  |  |
| *Sphingobacterium* |  |  |  |  | -0.30 |  |  |  |  |  |  |  |  |  |  |  |  |  |
| *Sporobacter* | -0.32 |  |  |  | 0.32 |  |  | 0.33 |  |  |  | -0.32 |  |  |  | 0.37 |  |  |
| *Streptococcus* |  |  |  |  |  |  |  |  | -0.30 |  |  |  |  |  |  |  |  |  |
| *Succinimonas* |  |  |  |  |  |  |  |  |  |  | -0.36 |  |  |  |  |  |  |  |
| *Succinispira* |  |  |  |  |  |  |  |  |  |  |  |  |  |  |  |  |  | -0.34 |
| *Succinivibrio* |  |  | 0.35 |  |  |  |  |  |  |  | -0.32 |  |  |  |  |  |  | 0.37 |
| *Treponema* |  |  | 0.33 |  |  |  |  |  |  |  |  |  |  |  |  |  |  | 0.31 |
| METHANOGENS COMUNITY |  |  |  |  |  |  |  |  |  |  |  |  |  |  |  |  |  |  |
| Concentration |  |  |  |  |  |  |  |  |  |  |  |  | 0.36 |  |  |  |  |  |
| Shannon Index |  |  |  | 0.30 |  |  |  |  |  |  |  |  |  |  |  |  |  |  |
| Family Methanobacteriaceae |  |  |  | 0.41 |  |  |  |  |  |  |  |  |  |  |  | 0.33 |  |  |
| Family Methanomassiliicoccaceae |  |  |  | -0.41 |  |  |  |  |  |  |  |  |  |  |  | -0.32 |  |  |
| *Methanobrevibacter* |  |  |  | 0.39 |  |  |  |  |  |  |  |  |  |  |  | 0.30 |  |  |
| *Methanobrevibacter gottschalkii* |  |  |  |  |  |  |  |  |  |  |  |  |  |  |  | 0.35 |  |  |
| *Methanobrevibacter ruminantium* |  |  |  | 0.47 |  |  |  |  |  |  |  |  |  |  |  |  |  |  |
| *Methanosphaera* | 0.31 |  |  | 0.53 |  |  |  |  |  |  |  |  |  |  |  | 0.40 |  |  |
| FUNGAL COMUNITY |  |  |  |  |  |  |  |  |  |  |  |  |  |  |  |  |  |  |
| Concentration |  |  | 0.38 |  |  |  | 0.52 |  | -0.37 |  |  |  | -0.33 |  |  |  |  |  |
| Richness |  |  | -0.39 |  | 0.33 |  | -0.54 |  | 0.36 |  | 0.32 |  | 0.41 |  |  |  |  |  |
| Shannon Index |  |  | -0.44 |  |  | 0.30 | -0.52 |  | 0.31 |  | 0.36 |  | 0.40 |  |  |  |  |  |
| Phylum Ascomycota |  |  | -0.37 |  |  | 0.35 | -0.55 |  | 0.43 |  |  |  | 0.51 |  |  |  |  |  |
| Phylum Basidiomycota |  |  | -0.46 |  | 0.38 |  | -0.57 |  | 0.41 |  | 0.36 |  |  |  |  |  |  |  |
| Phylum Neocallimastigomycota |  |  | 0.40 |  |  | -0.31 | 0.57 |  | -0.44 | -0.31 |  |  | -0.43 |  |  |  |  |  |
| *Buwchfawromyces* |  |  |  |  |  | 0.31 |  |  |  |  |  |  |  |  |  |  |  |  |
| *Caecomyces* |  |  | 0.34 |  |  |  | 0.44 |  |  | 0.39 | -0.33 |  | -0.38 | -0.31 |  |  |  | -0.47 |
| *Orpinomyces* |  |  | -0.40 |  |  | -0.36 | -0.37 | 0.35 |  | -0.46 |  |  |  |  |  |  |  | 0.37 |
| *Pecoramyces* |  |  | -0.37 |  |  |  | -0.32 |  |  | -0.39 |  |  |  |  |  |  |  | 0.35 |
| *Piromyces* |  |  | -0.35 |  |  |  |  |  |  |  |  |  |  |  |  |  |  |  |
| Yeast |  |  |  |  |  |  | -0.47 |  | 0.38 | 0.32 |  |  | 0.48 |  |  |  |  |  |
| PROTOZOAL COMUNITY |  |  |  |  |  |  |  |  |  |  |  |  |  |  |  |  |  |  |
| Concentration |  |  |  |  | 0.43 |  |  |  |  |  | 0.49 |  |  | 0.36 |  | 0.32 |  |  |
| Subfamily. Entodiniinae |  | -0.33 | -0.31 |  | 0.45 |  | -0.33 |  |  | 0.34 | 0.51 |  |  |  |  |  |  |  |

^1^Parameters: pH, ammonia-N (mg/dl), total volatile fatty acids (mM), Acetate (%), Propionate (%), Butyrate (%), Lactate (mM), Average daily gain (kg/d), Organic matter digestibility (%), in vitro methane (µmol/d). Microbial data was log transformed and only Spearman´s correlations coefficients >0.30 and *P*<0.01 were shown (n=72).

**Table S1.** Feed composition (in % of DM)

| **Feed** | **Concentrate** | **Ryegrass hay** | **Ryegrass pasture** | |
| --- | --- | --- | --- | --- |
| Organic matter | 92.6 | 93.6 | | 90.4 |
| Crude protein | 18.3 | 6.1 | | 11.4 |
| Neutral detergent fibre | 52.8 | 64.4 | | 51.0 |
| Acid detergent fibre | 13.9 | 34.6 | | 22.1 |

**Table S2.** Primers used for quantitative PCR and Ion-Torrent Next Generation Sequencing.

| **Target** | **Author** | **Forward Primer** | **Reverse Primer** | **T^a^** | | **Amplicon (bp)** |
| --- | --- | --- | --- | --- | --- | --- |
| **Quantitative PCR** |  |  |  | |  |  |
| Total bacteria | ([Maeda *et al.*, 2003](#_ENREF_4)) | GTGSTGCAYGGYTGTCGTCA | ACGTCRTCCMCACCTTCCTC | | 61 | 150 |
| Methanogens | ([Denman *et al.*, 2007](#_ENREF_2)) | TTCGGTGGATCDCARAGRGC | GBARGTCGWAWCCGTAGAATCC | | 56 | 140 |
| Anaerobic fungi | ([Denman & McSweeney, 2006](#_ENREF_1)) | GAGGAAGTAAAAGTCGTAACAAGGTTTC | CAAATTCACAAAGGGTAGGATGATT | | 62 | 120 |
| Protozoa | ([Sylvester *et al.*, 2004](#_ENREF_6)) | GCTTTCGWTGGTAGTGTATT | CTTGCCCTCYAATCGTWCT | | 55 | 223 |
| **Ion Torrent NGS** |  |  |  | |  |  |
| Bacterial primers | ([Spear *et al.*, 2008](#_ENREF_5)) | AGAGTTTGATCMTGGCTCAG | CTGCTGCCTYCCGTA | | 58 | 348 |
| Bacterial Adaptors |  | CCATCTCATCCCTGCGTGTCTCCGACTCAG | CCTCTCTATGGGCAGTCGGTGAT | |  |  |
| Methanogens primers | ([Wright & Pimm, 2003](#_ENREF_7)) | GCTCAGTAACACGTGG | GWATTACCGCGGCKGCTG | | 58 | 433 |
| Methanogens adaptors |  | CCATCTCATCCCTGCGTGTCTCCGACTCAG | CCTCTCTATGGGCAGTCGGTGAT | |  |  |
| Fungal primers | ([Detheridge *et al.*, 2016](#_ENREF_3)) | CYYAGTARCTGCGAGTGAAG | GAGCTG- CATTCCCAAACAA | | 52 | 200-220 |
| Fungal adaptors |  | CCATCTCATCCCTGCGTGTCTCCGAC | CCTCTCTATGGGCAGTCGGTGAT | |  |  |

**References**

Denman SE & McSweeney CS (2006) Development of a real-time PCR assay for monitoring anaerobic fungal and cellulolytic bacterial populations within the rumen. FEMS Microbiol Ecol 58: 572-582.

Denman SE, Tomkins N & McSweeney CS (2007) Quantitation and diversity analysis of ruminal methanogenic populations in response to the antimethanogenic compound bromochloromethane. FEMS Microbiol Ecol 62: 313-322.

Detheridge AP, Brand G, Fychan R, Crotty FV, Sanderson R, Griffith GW & Marley CL (2016) The legacy effect of cover crops on soil fungal populations in a cereal rotation. Agric Ecosys Environ 228: 49-61.

Maeda H, Fujimoto C, Haruki Y, Maeda T, Kokeguchi S, Petelin M, Arai H, Tanimoto I, Nishimura F & Takashiba S (2003) Quantitative real-time PCR using TaqMan and SYBR Green for *Actinobacillus actinomycetemcomitans , Porphyromonas gingivalis , Prevotella intermedia* , tetQ gene and total bacteria. FEMS Immunol Med Microbiol 39: 81 – 86.

Spear GT, Sikaroodi M, Zariffard MR, Landay AL, French AL & Gillevet PM (2008) Comparison of the diversity of the vaginal microbiota in HIV-infected and HIV-uninfected women with or without bacterial vaginosis. J Infec Dis 198: 1131-1140.

Sylvester JT, Karnati SKR, Yu ZT, Morrison M & Firkins JL (2004) Development of an assay to quantify rumen ciliate protozoal biomass in cows using real-time PCR. J Nutr 134: 3378-3384.

Wright ADG & Pimm C (2003) Improved strategy for presumptive identification of methanogens using 16S riboprinting. J Microbiol Meth 55: 337-349
